# LncRNA H19 as a key regulator in biliary atresia: insights into pathogenesis and potential therapeutic targets

**DOI:** 10.1080/07853890.2026.2654939

**Published:** 2026-05-14

**Authors:** Qi Zhao, Fujiao He, Zhibo Zhang, Lizhu Chen

**Affiliations:** a Department of Ultrasound, Shengjing Hospital of China Medical University, Shenyang, Liaoning, China; b Department of Pediatric Surgery, Shengjing Hospital of China Medical University, Shenyang, Liaoning, China

**Keywords:** LncRNA H19, biliary atresia, liver fibrosis, pathogenesis, therapeutic targets

## Abstract

**Background:**

Biliary atresia (BA) is a devastating cholangiopathy, characterized by onset of persistent cholestatic jaundice during the neonatal period. The etiology of BA remains incompletely understood and is considered multifactorial. Recent evidence has identified that the long non-coding RNA H19 (H19) is among the critical regulators involved in this pathological process. This review summarizes the current understanding of the H19 in the pathogenesis of BA.

**Discussion:**

The pathogenesis of BA is considered to be closely associated with impaired biliary epithelial barrier function. This barrier defect renders cholangiocytes more susceptible to injury and promotes bile acid retention. Within this pathological microenvironment, multiple factors such as bile acids and estrogen significantly upregulate H19 expression, with its level showing a positive correlation with the severity of BA. Up-regulated H19 is predominantly enriched in cholangiocytes and can be delivered to adjacent hepatocytes, hepatic stellate cells, macrophages, and liver sinusoidal endothelial cells, where it orchestrates cellular activities *via* diverse signaling pathways. By integrating and amplifying injury signals, H19 ultimately exacerbates inflammatory responses and fibrotic processes, driving the development and progression of BA. Additionally, emerging evidence suggests that certain traditional Chinese medicines exert anti-fibrotic effects by targeting H19, further highlighting its potential as a therapeutic target.

**Conclusion:**

This review comprehensively and systematically delineates the key regulatory network of H19 in BA, highlighting its translational potential as a novel therapeutic target and laying a theoretical foundation for future mechanistic exploration and clinical translation.

## KEY MESSAGES

1. LncRNA H19 is upregulated by multiple factors in the context of biliary atresia.
2. LncRNA H19 regulates diverse signaling pathways across different cell types.
3. LncRNA H19 acts as a target through which anti-fibrotic drugs exert their effects.

## 1. Introduction

Biliary atresia (BA) is a congenital cholangiopathy of unknown etiology in newborns, which is featured by inflammation and fibro-obliteration of biliary system [1], resulting in progressive cholestasis and severe liver fibrosis [2,3]. In clinical practice, BA presents as elevated direct bilirubin levels in the neonatal period, with persistent jaundice and pale stools [4]. Timely diagnosis and intervention are critical for improving clinical outcomes [5]. Currently, the Kasai portoenterostomy remains the primary surgical procedure to restore bile drainage [3]. However, the postoperative course is often complicated by recurrent cholangitis and progressive liver fibrosis [6], which may ultimately necessitate liver transplantation as a life-saving intervention [7,8].

The pathogenesis of BA has been reported in many studies, which mainly focus on the fields of genetic defects, environmental toxins, virus infection, epithelial-mesenchymal transition (EMT), matrix deposition, and decompensated angiogenesis [5,9,10]. In the signaling pathways involved in these biological processes, there is a growing focus on the regulatory role of non-coding RNAs (ncRNAs) at the upstream level. Although these RNA transcripts do not code for proteins, they play crucial roles in modulating intracellular physiological processes [11]. NcRNAs are broadly categorized based on the number of nucleotides. One category consists of short ncRNAs that are less than 200 bp, including microRNAs (miRNAs); another category is composed of RNA molecules longer than 200 bp, known as long ncRNAs (lncRNAs) [12]. MiRNAs are able to induce translational inhibition or degradation of target mRNAs by binding to the complementary sequences in the 3′ untranslated region, thereby regulating the expression of target genes at the post-transcriptional level [13]. However, lncRNAs can absorb miRNAs as competitive endogenous RNA (ceRNA) and interfere with the regulation of miRNAs on the expression of target genes [14].

LncRNA H19 (H19) is a paternally imprinted and maternally expressed gene [15], which is highly expressed during the fetal period, down-regulated after birth, with the exception of skeletal muscle [16]. H19 is the first lncRNA to be identified and studied in the liver [12]. There are also elevated levels of H19 expression in liver diseases with various etiologies, encompassing nonalcoholic fatty liver disease, cholestatic liver disease, hepatitis, liver fibrosis, and hepatocellular carcinoma [14,16–18]. Moreover, the potential of H19 as a therapeutic target for liver diseases has been comprehensively explored in the literature [19]. In the context of BA, H19 is transported *via* exosomes among various cell types, including cholangiocytes, hepatocytes, hepatic stellate cells (HSCs), and macrophages, thereby coordinating the physiological interactions between these cells [20–24].

This review focuses on the role of H19 in BA pathogenesis. Furthermore, it summarizes evidence that the anti-fibrotic efficacy of several drugs is mechanistically linked to H19-dependent pathways, setting a framework for further exploration.

## 2. Pathological features of BA

Recent studies have increasingly implicated defective embryogenesis of the liver and biliary tract as a potential contributing factor to the pathogenesis of BA [5,25]. Consistent with that, severely disrupted cell junctions and polarity complexes [26,27], as well as abnormal ciliogenesis [28,29], have been observed in BA. Additionally, the presence of abnormal inflammatory and innate immune responses also has been proposed as potential etiologies [30,31]. Therefore, it can be reasonably speculated that increased permeability and susceptibility to injury exist in cholangiocytes due to a poor development [32]. When cholangiocytes are attacked by external or internal invading factors, even slight injuries can propagate into the subepithelial compartment, further expanding the degree of epithelial damage [32]. Bile acids accumulate rapidly within this inappropriate immune microenvironment, inducing the production of various pro-inflammatory and pro-fibrotic factors, among which H19 is a notable example. In turn, increasing levels of H19 aggravates the immune injury response, which contributes to the obliteration of the lumen, and further impedes bile acid flow and initiates the occurrence of BA.

## 3. The expression mechanisms of H19

H19, a paternally imprinted and maternally expressed gene, resides close to the telomeric region of chromosome 11p15.5 in homo sapiens, downstream of another imprinted gene, insulin-like growth factor 2 (Igf2) [33]. The expression of H19 is controlled by a promoter, distal enhancers and an imprinting control region (ICR). The CCCTC binding factor (CTCF) binds to the ICR and modulates this regulatory process, enhancing H19 transcription [34,35]. Notably, H19 is the precursor of miRNA675 (miR-675), which is a highly correlated miRNA that is located within exon-1 of the H19 gene [36].

In cases of BA, it has been documented that H19 is primarily up-regulated by bile acids and is predominantly expressed in cholangiocytes [20–22], which aligns well with the pathological features of BA. Furthermore, a positive correlation was observed between the magnitude of the increase in H19 expression and the severity of the disease [24]. It is widely recognized that both conjugated bile acids and estrogen are involved in the pathogenesis and progression of cholestatic liver diseases and cholangiopathies [37,38]. Consistent with that, both taurocholate acid (TCA)-mediated activation of sphingosine-1-phosphate receptor 2 (S1PR2) and estrogen-induced activation of estrogen receptor jointly activate the extracellular signal-regulated kinase (ERK) 1/2 pathway, thereby up-regulating H19 expression in cholangiocytes [20]. A significant gender difference in H19 upregulation was also observed, with female multidrug resistance gene 2 knockout (Mdr2-/-) mice exhibiting more pronounced liver and bile duct injury than males. The knockdown of H19 can markedly ameliorate the cholestatic injury in female mice [20]. This highlights a promising research direction for exploring the mechanistic interplay between estrogen signaling and BA pathogenesis. Moreover, TCA and estrogen not only elevate the levels of H19 in cholangiocytes, but also result in an augmentation of exosomes size and quantity [21]. H19 is delivered into hepatocytes *via* exosomes, modulating hepatocytes activities through various pathways and ultimately giving rise to injury to hepatocytes [20,21]. Besides, exosomes containing H19 are also transferred to other cells, such as liver Kupffer cells (KCs) [23] and HSCs [22], leading to their activation and proliferation.

**Figure 1.**
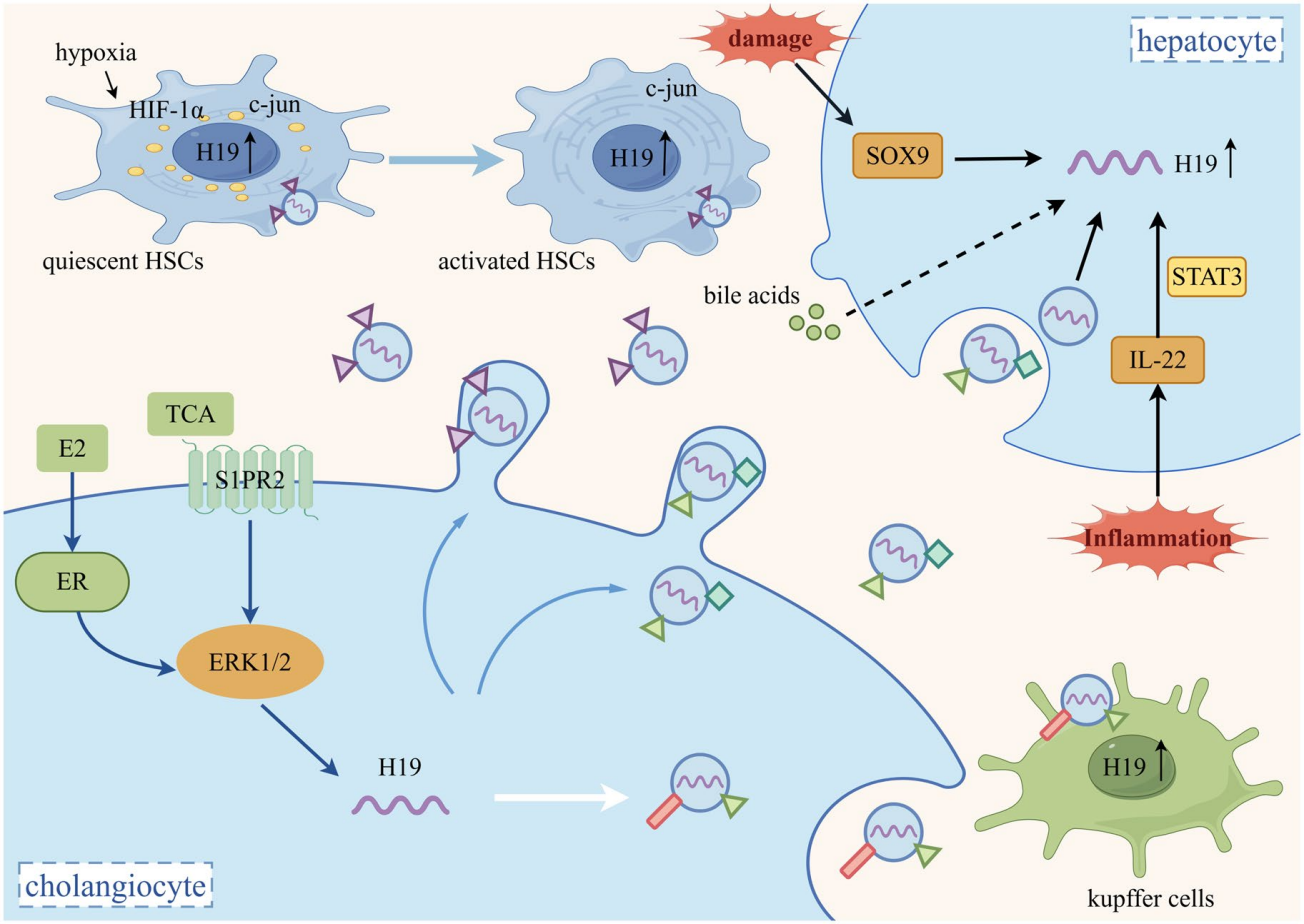
Regulation of H19 expression and secretion (by Figdraw). TCA -mediated activation of S1PR2 and estrogen-induced activation of estrogen receptor trigger the activation of ERK1/2 pathway, leading to up-regulation of H19 in cholangiocytes, which is the main source of H19. H19 is transported from cholangiocytes to hepatocytes, HSCs and KCs by a form of exosome. In hepatocytes, H19 is up-regulated by bile acids, IL-22 and SOX9. In HSCs, the transcription of H19 is promoted by HIF-1α and c-jun. In Kupffer cells, H19 is also up-regulated through some autocrine pathways. TCA: taurocholate acid; S1PR2: sphingosine-1-phosphate receptor 2; E2: estrogen; ER: estrogen receptor; ERK1/2: extracellular signal-regulated kinase 1/2; SOX9: sex-determining region Y (SRY)-box 9; IL-22: Interleukin-22; STAT3: signal transducer and activator of transcription 3; HSCs: hepatic stellate cells; HIF-1α: Hypoxia-inducible factor-1α.

Nevertheless, the increased H19 in hepatocytes, KCs, and HSCs does not exclusively originate from exosomes released by cholangiocytes. A fraction of H19 is produced through the autocrine pathway within these cells under the regulation of other factors, including but not limited to inflammatory stimuli and hypoxic conditions. Similar to cholangiocytes, a part of H19 in hepatocytes is produced in response to excessive bile acids [39]. Other inducers such as Interleukin-22 (IL-22) [40] and sex-determining region Y (SRY)-box 9 (SOX9) [41] can also elevate the expression of H19 under the condition of hepatocytes injury. During the activation of HSCs, hypoxia-inducible factor-1α (HIF-1α) increases and promotes the transcription of H19 by binding to the hypoxia response element site on its promoter [42]. Furthermore, a new regulatory cascade response consisting of c-Jun/H19/miR-19a/b-3p/c-Jun N-terminal kinases 1 (JNK1)/c-Jun has been illustrated during HSCs activation, which points out a new mechanism of HSCs activation and transition from the initial activation state to the permanent activation state [43]. Additionally, H19 is also up-regulated in CD11b + monocytes/macrophages and F4/80+ macrophages in the livers of bile duct ligation (BDL) mice models [44]. Notably, the expression levels of H19 in cholangiocytes are approximately 100 times greater than in hepatocytes, HSCs, and KCs [22] in BDL and Mdr2-/- mice models. Therefore, the release of H19 by cholangiocytes represents the primary origin of elevated H19 levels in BA. The expression of H19 in these cells is shown in Figure 1.

## 4. The regulation targets of H19

The bile duct obliteration and liver fibrosis present in BA are dynamic and evolving. Depending on the nature and severity of the injury, parenchymal cells and non-parenchymal cells are activated to differing degrees, and subsequently proliferate due to their capability to communicate through various paracrine and autocrine signals. During this process, H19 contributes to the response of these cells to injury, which not only promotes the proliferation of parenchymal cells, but also induces the activation and proliferation of non-parenchymal cells. Moreover, H19 can be transported among these cells in the form of exosomes, thereby serving as a bridge for intercellular communication that coordinates and motivates the advancement of BA.

### 4.1. Parenchymal cells

#### 4.1.1. Cholangiocytes

Recent studies have shown that H19 promotes cholangiocytes proliferation and liver fibrosis by modulating the S1PR2/Sphingosine kinase 2 (SphK2) and miRNA let-7 (let-7)/high mobility group protein AT-hook 2 (HMGA2) signaling pathways [24]. S1PR2 is a receptor expressed in cholangiocytes and hepatocytes, which can be activated and its expression levels can be elevated by TCA in a dose-dependent approach under conditions of bile stasis [45]. Subsequently, TCA/S1PR2 mediates the activation of ERK1/2 signaling pathways, leading to inflammation and proliferation of cholangiocytes, and the knockout of S1PR2 in mice results in protection from these effects [45]. SphK2 functions as the downstream mediator of S1PR2, catalyzing the phosphorylation of sphingosine to generate sphingosine 1 phosphate (S1P), which serves as the ligand for S1PR2 to induce its activation, analogous to TCA [46]. What’s more, the levels of H19 are significantly up-regulated in BA patients, showing a positive correlation with the expression of S1PR2 and SphK2, as well as severity of liver injuries [24]. The deficiency of H19 markedly blocks the elevation of TCA-induced S1PR2 expression, which inhibits bile duct proliferation and liver fibrosis by attenuating S1PR2/SphK2 signaling [24].

HMGA2 is a known negative regulatory target of let-7 and promotes biliary proliferation. H19 functions as a molecular sponge for members of the let-7 family to regulate let-7/HMGA2 axis [24]. Additionally, it has also been indicated that H19 suppresses the availability but promotes the biosynthesis of let-7 by interacting with polypyrimidine tract binding protein 1 (PTBP1) in cholestasis [47]. PTBP1 is an RNA-binding protein and regulates precursor mRNA (pre-mRNA) splicing, which can bind with pre-let-7 to suppress the maturity of let-7. However, PTBP1 is negatively regulated by H19 [48]. Hence, this article provides a new insight about the regulation of H19 on let-7 in detail.

It has been demonstrated that H19 promotes cholestatic liver fibrosis in mice through the zinc finger E-box binding homologous protein 1 (ZEB1)/epithelial cell adhesion molecule(EpCAM) signaling pathway [39]. ZEB1 is a transcriptional repressor that triggers EMT during fibrosis [48]. EpCAM, also referred to as CD326, is associated with enhanced cell proliferation [49]. Mechanistically, H19 interacts with the ZEB1 protein to prevent its binding to the EpCAM promoter, thereby alleviating the suppression effect of ZEB1 on EpCAM expression. Consequently, H19 demonstrates an activating effect on EpCAM, further promoting cell proliferation and fibrosis [39]. Interestingly, the expression of H19 is more prominently up-regulated in small cholangiocytes compared to large cholangiocytes [39]. It is worth noting that small cholangiocytes show a high nucleo-cytoplasmic ratio, indicating their classification as hepatic progenitor cells (HPCs) [50], which implies that H19 may also contribute to fibrosis progression by modulating HPCs [39].

**Figure 2.**
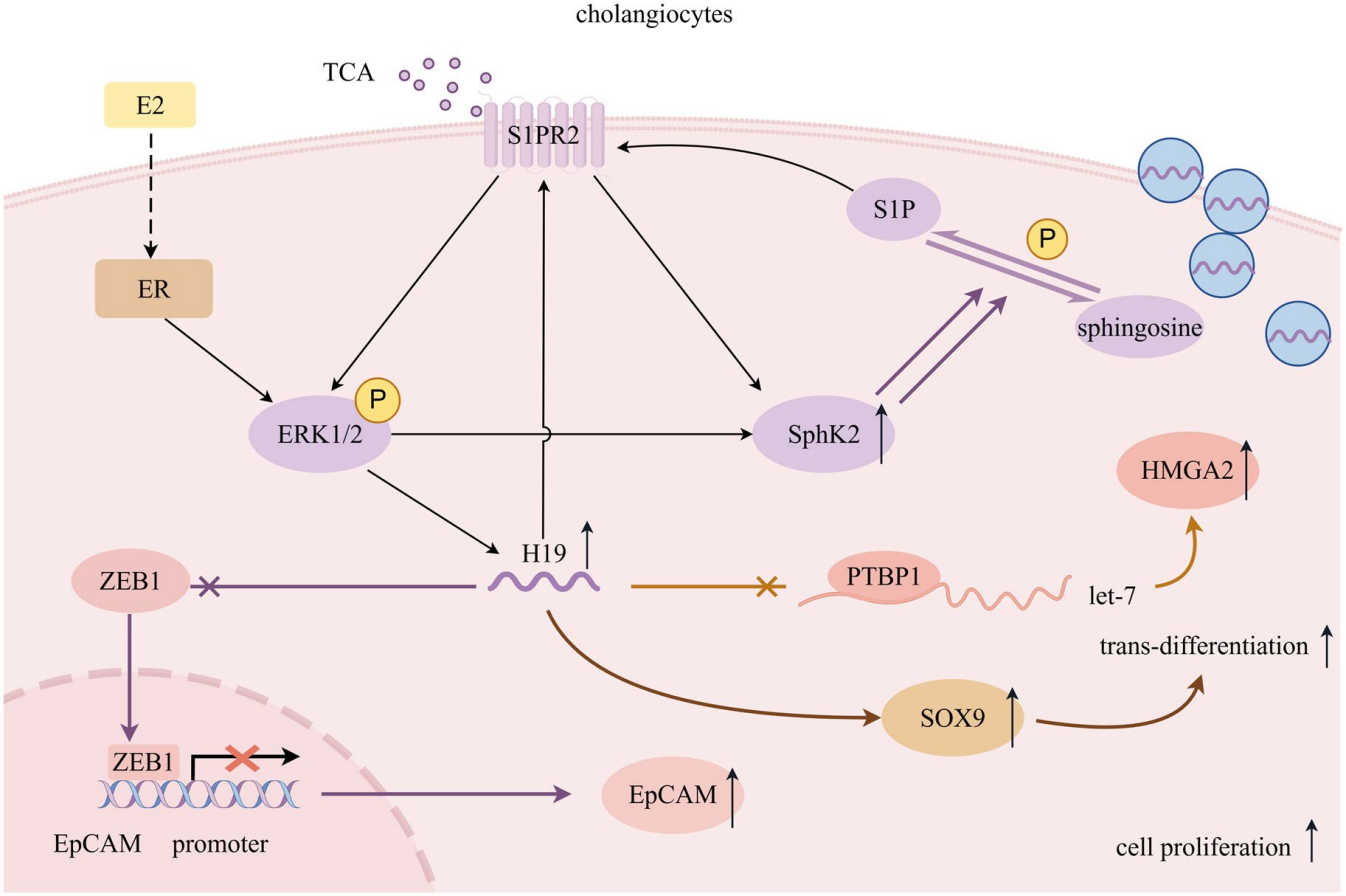
Potential targets of H19 regulated in cholangiocytes (by Figdraw). The activation of ERK1/2 pathway promotes the expression of H19 and Sphk2. S1PR2 regulates the expression and function of SphK2, increased H19 promoted the S1PR2/SphK2 axis. SphK2 phosphorylates sphingosine to produce S1P, which is also an activator of S1PR2. H19 inhibits the bioavailability of let-7 by negatively regulating PTBP1, up-regulating the effect of HMGA2. H19 hinders the binding of ZEB1 to the EpCAM promoter, relieves the inhibitory effect of ZEB1 on EpCAM, promoting the increase of EpCAM expression. H19 increases the expression of SOX9, which may be helpful for trans-differentiation and proliferation of cholangiocytes. E2: estrogen; ER: estrogen receptor; TCA: taurocholate acid; S1PR2: sphingosine-1-phosphate receptor 2; ERK1/2: extracellular signal-regulated kinase 1/2; Sphk2: Sphingosine kinase 2; S1P: sphingosine 1-phosphate; PTBP1: polypyrimidine tract binding protein 1; HMGA2: high mobility group protein AT-hook 2; ZEB1: zinc finger E-box binding homologous protein 1; EpCAM: epithelial cell adhesion molecule; SOX9: sex-determining region Y (SRY)-box 9.

The proliferation of cholangiocytes is recognized as reactive bile duct hyperplasia after damage, which is pathologically defined as ductular reaction (DR) [51]. The cells involved in DR are referred to as reactive ductal cells (RDCs), which can arise from a range of cell types, such as mature cholangiocytes, HPCs, or periportal hepatocytes [51,52]. It has been clarified that HPCs within portal triads and adjacent to biliary ductules contribute to the majority of newly differentiated cholangiocytes in BA [53,54]. In some studies, EpCAM and SOX9 have frequently served as markers for identifying HPCs [51]. Consistently, elevated levels of SOX9 are also observed in instances of H19 over-expression [39]. Notably, the aberrant expression of SOX9 has been identified as closely associated with the trans-differentiation and proliferation of HPCs, thus playing a pivotal role in BA [55]. The aforementioned findings further reinforce the hypothesis that H19 is capable of modulating the activities of HPCs in BA. The aforementioned impacts of H19 on cholangiocytes are outlined in Figure 2.

#### 4.1.2. Hepatocytes

Echoing the previous conclusion, it has been confirmed that liver progenitor-like cells (LPLCs) originating from mature hepatocytes are also a type of RDCs and closely associated with the progression and prognosis of BA [56]. LPLCs, which present as bipotent progenitors, have been identified through SOX9 and hepatocyte nuclear factor 4α (HNF4α) markers [56]. As disease advances, SOX9 is re-upregulated in hepatocytes, causing a phenotypic switch in them and reprogramming into LPLCs [57]. In instances of hepatocytes damage, SOX9 also induces higher levels of H19 in a certain way [41]. Consistent with this, double-positive H19+/SOX9+ ductal progenitor cells, H19+/HNF4α+ hepatocytes, as well as triple-positive H19+/HNF4α+/SOX9+ periportal hepatocytes have been identified in cholestatic livers [58]. The aforementioned findings provide convincing evidence that H19 can promote DR by impacting RDCs, thereby contributing to the advancement of fibrosis in BA.

It was indicated that B-cell lymphoma protein 2 (Bcl2) was reactivated within the context of liver injury, leading to the degradation of the small heterodimer partner (SHP) protein through the activation of the caspase8 pathway [59]. SHP has been recognized as a transcriptional repressor. Nevertheless, following its down-regulation, the inhibitory influence of SHP on H19 expression was mitigated, leading to a prominent increase in H19 expression [59]. Unexpectedly, this initial finding was later contested, as subsequent research demonstrated that there was no association between Bcl2 and H19-induced liver injury [20]. Additionally, the article supported the presence of a reverse correlation between H19 and SHP, where H19 functioned as a controlling factor that influenced SHP [20]. Subsequently, the regulatory mechanism of H19 on SHP was further elucidated in a separate publication. It was validated that exosomal H19 derived from cholangiocytes acted on hepatocytes and suppressed SHP expression by inhibiting its promoter activity and destabilizing mRNA [21]. SHP is a nuclear receptor that played a crucial role in maintaining bile acid homeostasis [60]. Hence, the down-regulation of SHP mediated by H19 resulted in a disruption in bile acid homeostasis, thereby potentiating the extent of liver damage, which formed a vicious circle. Besides, the activation of ERK1/2 mediated by TCA and estrogen in hepatocytes can also suppress SHP expression through ERK1/2/liver kinase B1 (LKB1)/AMP-activated protein kinase (AMPK) axis [38].

Furthermore, H19 serves as the crucial precursor molecule for the biogenesis of miR-675, wherein the up-regulation of H19 is accompanied by a consequential increase in the expression levels of miR-675 [37]. Fas-associated protein with death domain (FADD), a key mediator within the apoptotic signaling cascade, undergoes negative regulation at the post-transcriptional level by miR-675 [62]. Thus, H19 exerts its inhibition on FADD by augmenting miR-675 levels, consequently promoting necroptosis in hepatocytes and exacerbating inflammatory responses in liver [61].

In contrast to the aforementioned findings, other research has demonstrated that H19 has the partial effect of protecting the liver from damage. In liver damage, the elevation of IL-22 markedly stimulates the expression of H19 in hepatocytes, a process that is dependent on signal transducer and activator of transcription 3 (STAT3) for modulation [40]. IL-22 regulates the activation of AMPK signaling pathway to ameliorate mitochondrial energy metabolism disorders, a process that relies on the presence of H19 [41]. H19 modulates AMPK/protein kinase B (AKT)/mammalian target of rapamycin (mTOR) axis, which in turn inhibits the buildup of reactive oxygen species in liver cell mitochondria and promotes the clearance of damaged mitochondria following liver injury, exerting a protective effect [40].

**Figure 3.**
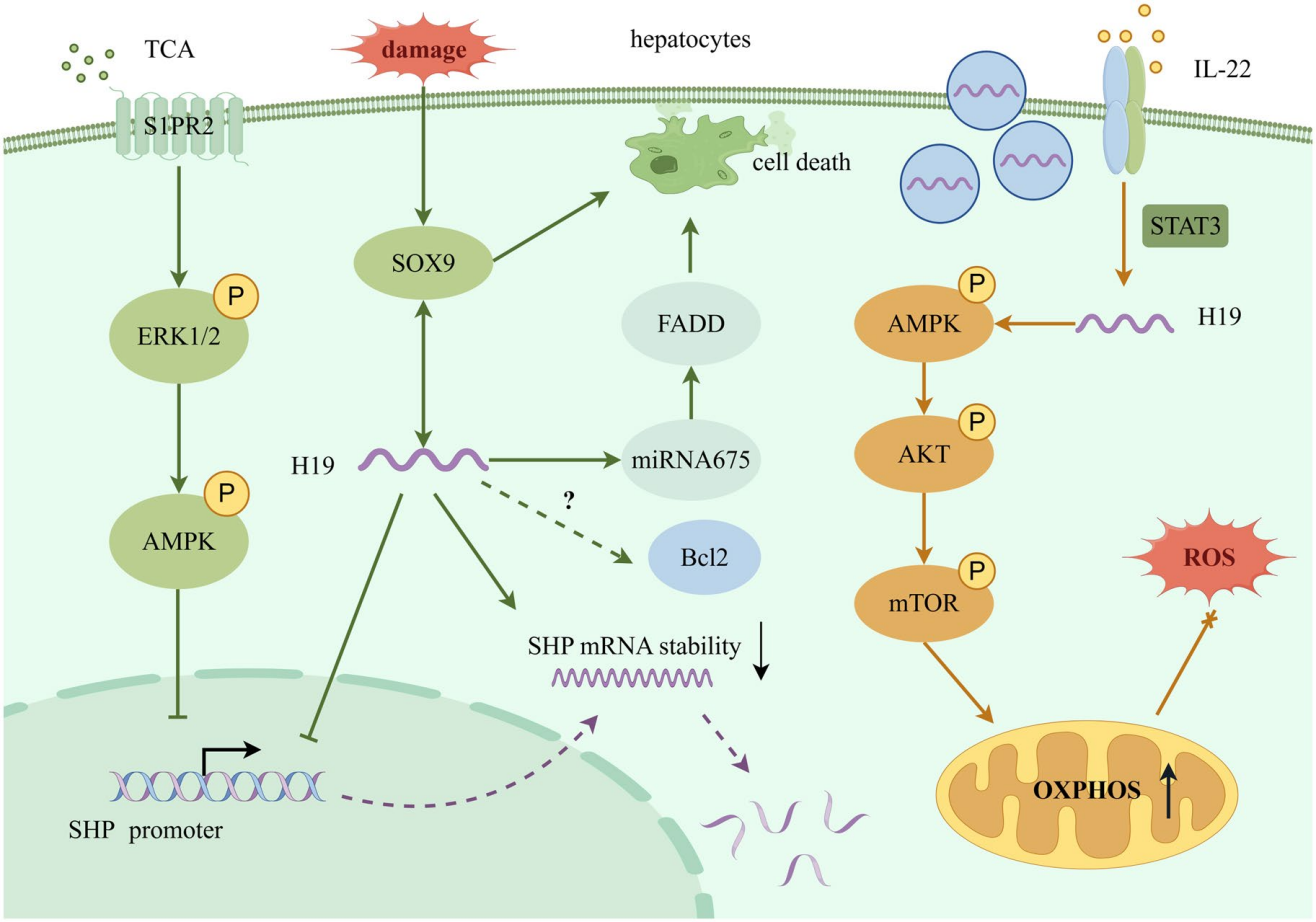
Potential targets of H19 regulated in hepatocytes (by Figdraw). TCA induces the activation of S1PR2/ERK1/2/AMPK axis, inhibiting the expression of SHP. H19 suppresses the expression of SHP by reducing promotor activity and destabilizing SHP mRNA. The role of H19 and Bcl2 remains unknown and needs to be further verified. The increase of SOX9 in the case of injury up-regulates H19 level, which may be bidirectional. SOX9/H19 axis may induce cell death or trans-differentiation. H19 is a precursor of miRNA675, increased miRNA675 targets FADD to promote cell death. H19 modulates the AMPK/AKT/mTOR pathway to elevate OXPHOS and clear ROS under the induction of IL-22. TCA: taurocholate acid; S1PR2: sphingosine-1-phosphate receptor 2; ERK1/2: extracellular signal-regulated kinase 1/2; SHP: small heterodimer partner; Bcl2: B-cell lymphoma protein 2; SOX9: sex-determining region Y (SRY)-box 9; FADD: Fas-associated protein with death domain; IL-22: Interleukin-22; STAT3: signal transducer and activator of transcription 3; AMPK/AKT/mTOR: AMP-activated protein kinase/protein kinase B/mammalian target of rapamycin; ROS: reactive oxygen species; OXPHOS: oxidative phosphorylation.

While the conclusions reached by different studies may vary, this discrepancy highlights the intricate nature of the function of H19, underscoring the necessity for further research to investigate and refine the H19 regulatory network. The aforementioned impacts of H19 on hepatocytes are outlined in Figure 3.

### 4.2. Non-parenchymal cells

#### 4.2.1. Hepatic stellate cells

EMT is a basic pathophysiological phenomenon, which is marked by the conversion of E-cadherin expression to N-cadherin in polarized epithelial cells to acquire the mesenchymal cell phenotype [62]. Recent research has found that EMT is observed in cholangiocytes, hepatocytes, and HSCs, leading to characteristics with bile duct proliferation, obliteration and liver fibrosis in BA [9]. H19 drives the conversion of HSCs into myofibroblasts by activating some pathways associated with EMT, thereby contributing to the progression of fibrosis.

A recent study has revealed that H19 epigenetically regulates the reprogramming of lysine 27 trimethylation on histone 3 (H3K27me3) profiles of genes involved in cell proliferation and extracellular matrix deposition by interacting with enhancer of zeste homolog 2 (EZH2) directly [63]. This process induces the activation of Wnt/β-catenin signaling pathway, which triggers the EMT and proliferation of HSCs. EZH2 can catalyze the trimethylation of H3K27 by acting as a key catalytic protein that belongs to polycomb repressive complex 2 and contributes to silencing gene expression [64]. β-catenin is a key nuclear effector of canonical Wnt signaling, which is significantly induced by increased H19. Consistently, the distribution of H3K27me3 peaks on its gene is markedly decreased [63].

Furthermore, H19 acts as a ceRNA for miRNA148a, facilitating the EMT and the activation of HSCs through the H19/miRNA148a/ubiquitin-specific protease 4 (USP4)/transforming growth factor β (TGF-β) axis [65]. USP4, a known negative target of miRNA148a, has the ability to enhance the stability of the TGF-β receptor I [66]. By acting as a ceRNA to sponge miR-148a, H19 leads to enhanced USP4 function, which ultimately activates the TGF-β signaling pathway.

The activation of HSCs and the subsequent series of physiological activities require a large amount of energy [67]. Research has suggested that this energy is derived from the degradation of lipid droplets (LDs) and the lipid oxidation stored in quiescent HSCs [68]. The existence of LDs is a notable feature in quiescent HSCs [69], which consist of retinyl esters (the storage form of vitamin A), triglycerides, and cholesterol esters [70]. The dramatic disappearance of LDs signifies the activation of HSCs [70], whereas the restoration of LDs has the potential to alleviate liver fibrosis [71]. Several studies provide evidence for the hypothesis that H19 is capable of regulating LD metabolism to support the transition of HSCs into myofibroblasts.

HIF-1α has been shown to elevate H19 levels in HSCs. The elevated H19 serves as a scaffold to connect LKB1 with AMPK to form a complex that promotes LKB1-mediated AMPK phosphorylation [42]. AMPK is activated through phosphorylation, leading to the initiation of various metabolic pathways including downstream LDs degradation and lipid oxidation, ultimately facilitating the transformation of HSCs into myofibroblasts [42]. Upon H19 knockdown, the expression of peroxisome proliferator-activated receptor α (PPARα), a key player in lipid oxidation, and its target gene, Carnitine Palmitoyltransferase 1 A (CPT1A), is significantly down-regulated. In contrast, the expression levels of genes associated with lipid uptake and synthesis remain largely unchanged [42].

However, H19-induced AMPK signaling can be suppressed by dihydroartemisinin (DHA) [72]. DHA down-regulates the expression of HIF-1α by activating the PI3K/AKT pathway, which leads to an obvious attenuation of HIF-1α-mediated up-regulation of H19 transcription. Consistent with the aforementioned findings, the administration of DHA leads to a marked down-regulation of the expression levels of PPARα and CPT1A [72]. Therefore, DHA inhibits LDs depletion *via* the HIF-1α/H19/AMPK signaling cascade, thereby suppressing HSC activation and ameliorating liver fibrosis.

The metabolism of retinol in LDs is crucial for the activation of HSCs, and the disappearance of LDs lacking retinol does not aggravate the severity of liver fibrosis [73]. It is widely acknowledged that retinol esters are hydrolyzed to produce retinol, which could be further oxidized by alcohol dehydrogenases (ADHs) and retinaldehyde dehydrogenases (ALDHs) to form retinoic acid (RA) eventually [74]. H19 enhances the activation of HSC, which is also attributed to its up-regulation of ADH III-mediated RA signals [75]. RA interacts with diverse nuclear receptors such as retinoid A receptors and retinoid X receptors, leading to the up-regulation of gene expression of various inflammation factors like monocyte chemotactic protein-1 and Interleukin 6, as well as fibrotic factors, such as collagen type I and α-smooth muscle actin, which aggravate the inflammation and fibrosis [75]. Similarly, DHA treatment also relieves liver fibrosis through impairing H19-mediated RA signals [75].

**Figure 4.**
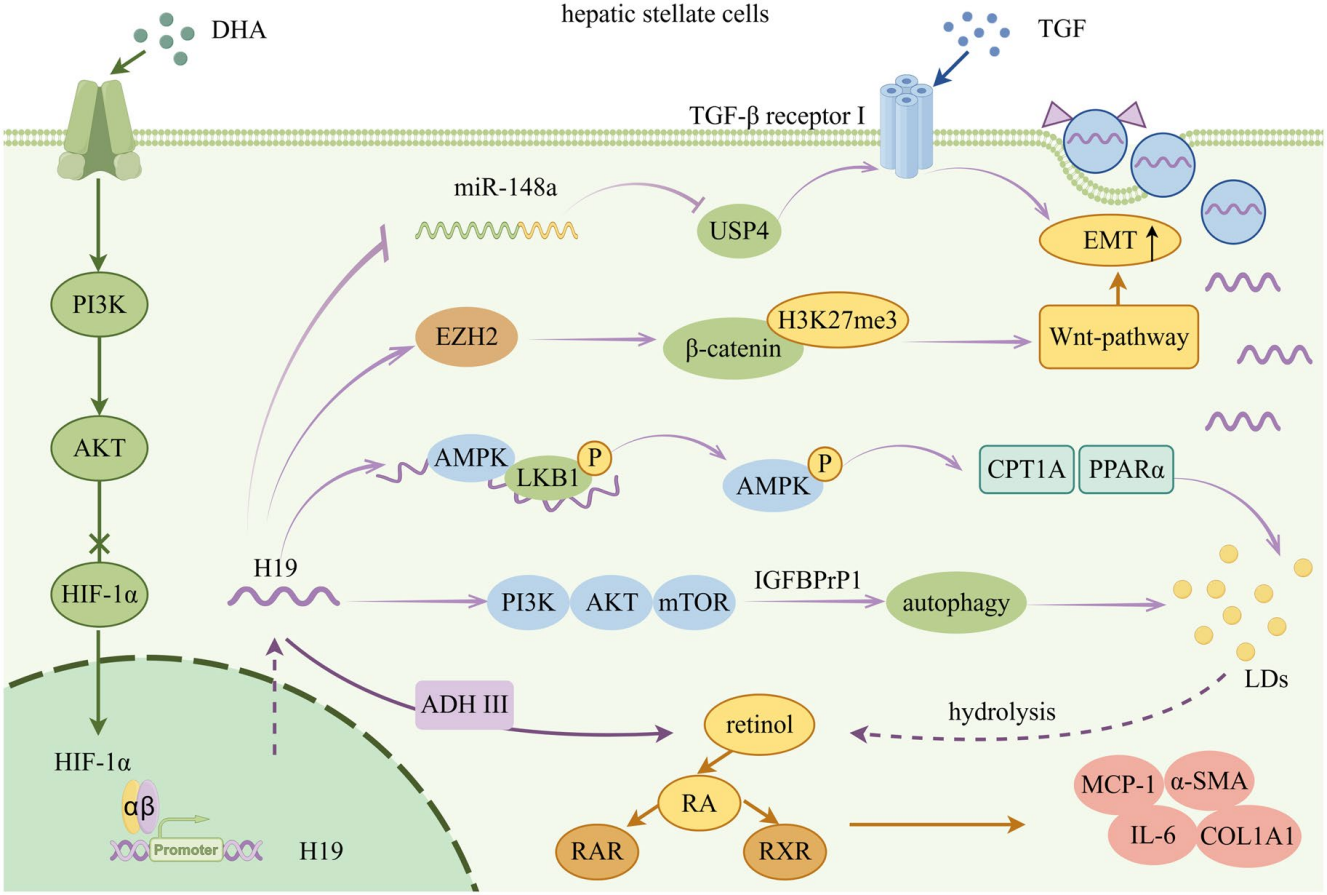
Potential targets of H19 regulated in HSCs (by Figdraw). H19 acts as the ceRNA of miRNA148a, competing with it to bind to USP4, promoting the TGF β1pathway. H19 binds to EZH2 and reprograms the H3K27me3 profile of genes involved in cell proliferation and Wnt pathway. H19 serves as a scaffold to promote the formation of LKB1/AMPK complex and subsequent AMPK activation, inducing the expression of CPT1A and PPARα. H19 regulates PI3K/AKT/mTOR axis to participate in IGFBPrP1-induced autophagy. H19 facilitates retinoic acid signal pathway and up-regulates the secretion of pro-inflammatory and pro-fibrotic factors by interacting with ADH III. DHA inhibits the expression of HIF-1α by activating PI3K/AKT pathway, thereby impeding the up-regulation of H19. DHA: dihydroartemisinin; PI3K/AKT/mTOR: phosphoinositide 3-kinase/protein kinase B/mammalian target of rapamycin; HIF-1α: Hypoxia-inducible factor-1α; ceRNA: competitive endogenous RNA; USP4: ubiquitin-specific protease 4; TGF: transforming growth factor; EMT: Epithelial-mesenchymal transition; EZH2: enhancer of zeste homolog 2; H3K27me3: lysine 27 tri-methylation on histone 3; AMPK: AMP-activated protein kinase; LKB1: Liver kinase B1; CPT1A: Carnitine Palmitoyltransferase 1A; PPARα: peroxisome proliferator-activated receptor α; LDs: lipid droplets; IGFBPrP1: Insulin-Like Growth Factor Binding Protein-Related Protein 1; ADH III: alcohol dehydrogenases III; RA: retinoic acid; RAR: retinoid A receptor; RXR: retinoid X receptor; MCP-1: monocyte chemotactic protein-1; IL-6: Interleukin 6; COL1A1: collagen type I; α-SMA: α-smooth muscle actin.

H19 also possesses the ability to indirectly regulate LDs metabolism by facilitating autophagy. It has been elucidated that autophagy can drive the activation of HSCs by generating fatty acids from the cleavage of retinyl esters within LDs [68–70]. The PI3K/Akt/mTOR signaling pathway is the primary pathway for regulating autophagy [76]. It has been observed that the expression of Insulin-Like Growth Factor Binding Protein-Related Protein 1 (IGFBPrP1) significantly increases in BDL-induced fibrosis, concurrently with the increase of H19 [77]. H19 interacts with the PI3K/Akt/mTOR signaling pathway to promote autophagy, which in turn contributes to the IGFBPrP1-induced activation of HSCs [77]. The underlying significance of the IGFBPrP1/H19/PI3K/AKT/mTOR/autophagy pathway in liver fibrosis warrants further investigation to provide new insights into the therapeutic strategies. The aforementioned impacts of H19 on HSCs are outlined in Figure 4.

#### 4.2.2. Macrophages

Hepatic macrophages, comprising resident KCs and recruited bone marrow-derived monocyte/macrophages, play a crucial role in BA [78]. The previous studies have shown that increased macrophage infiltration in BA patients is strongly correlated with damage to cholangiocytes [79], which facilitates the advancement of fibrosis even following KPE treatment [80].

Cholangiocyte-derived H19-carrying exosomes can be rapidly internalized by KCs, leading to a significant activation of KCs in cholestatic liver disease [23]. Simultaneously, H19 up-regulates the expression and secretion of chemokine (C–C motif) ligand 2 and IL-6 in KCs, thereby promoting the recruitment and differentiation of bone marrow-derived monocyte/macrophages, which deteriorates the inflammation and fibrosis [23]. Furthermore, it has been demonstrated that selective depletion of macrophages effectively suppresses liver fibrosis and cholangiocyte proliferation. Conversely, H19 overexpression counteracts these effects, thus identifying it as a pivotal regulator of macrophage function [44]. In summary, H19 produced by the autocrine pathway mediates cholangiocyte proliferation and liver fibrosis by regulating macrophage activity [44].

#### 4.2.3. Liver sinusoidal endothelial cells

It has been indicated that H19 participates in hypoxia stress in liver sinusoidal endothelial cells (LSECs) by promoting NADPH oxidases (NOX4) and inhibiting endothelial NOS (eNOS) signaling pathway [81]. NOX4 mediates hepatocyte death and HSC activation during liver fibrosis, whereas eNOS signaling exert an opposite effect. MiR-148b-3p negatively regulates NOX4 and positively modulates eNOS, while H19 acts as a ceRNA that sponges miR-148b-3p [81]. Therefore, H19 negates the modulation of miR-148b-3p on these two signaling pathways, which indirectly regulates the activity of LSECs and enhances their response to hypoxia, thereby facilitating their involvement in fibrosis [81].

In the setting of liver injury, LSECs undergo phenotypic transformation, characterized by the disappearance of fenestrations and the emergence of a subendothelial basement membrane, a phenomenon referred to as capillarization [82]. The significant structural alteration fosters extracellular matrix deposition, thereby increasing resistance to blood flow and triggering micro-circulation disorders. Consequently, these changes further aggravate the progression of fibrosis, establishing a vicious cycle [82]. Vascular endothelial growth factor (VEGF) derived from hepatocytes and HSCs is the most critical regulator for LSECs phenotype [82]. It has been demonstrated that elevated VEGF expression in patients with BA contributes to the progression of biliary and hepatic fibrosis [83–85]. What’s more, HIF-1α becomes activated in cholangiocytes in a state of hypoxia-ischemia, stimulating the secretion of VEGF and modulating its biological activities within the biliary structures of patients with BA [86]. The transcription of H19 has been verified to be promoted by HIF-1α [42], suggesting that it may function as a key mediator in the HIF-1α-mediated pathological processes of BA. However, the precise mechanisms remain elusive and necessitate further investigation of the correlation between H19 and VEGF in the future.

## 5. H19-targeting herbal therapy

Due to the prominent role that H19 plays in liver diseases, current research have explored pharmaceutical treatments that focus on targeting H19 to ameliorate hepatic fibrosis, primarily involving DHA [73,76] and various Traditional Chinese Medicines, such as Chuanxiong Rhizoma (CX) extracts [87], Si-Wu-Tang (SWT) [88], and Danhongqing formula (DHQ) [89].

### 5.1. Chuanxiong Rhizoma

CX is a kind of Traditional Chinese Medicine. Its extracts down-regulate the expression of H19 *via* inhibiting the CTCF-c-MYC-H19 pathway both *in vivo* and *in vitro*, which significantly suppress diffuse severe bile duct hyperplasia induced by bile acids and TGF [87]. It has been confirmed that c-MYC possesses the capability to enhance the transcriptional activity of H19. Meanwhile, the ICR of H19 and the promoter region of c-MYC contain CTCF binding sites, which allow CTCF to regulate the transcription of both H19 and c-MYC [90]. Different kinds of CX extracts down-regulate the expression of c-MYC and CTCF to varying degrees, thereby demonstrating a significant decrease in H19 expression [87]. Moreover, another study has demonstrated that senkyunolide A (Sen A), a bioactive extract isolated from CX, exerts its antifibrotic effects by inhibiting cholangiocyte proliferation through targeting the c-MYC/H19/let-7a signaling axis, in which H19 plays a central regulatory role [91].

Notably, CX extracts also inhibit the combination of lysine acetyltransferase 2 A (KAT2A) and mothers against decapentaplegic homolog 3 (SMAD3) to down-regulate the histone 3 lysine 9 acetylation (H3K9ac), which subsequently decreases the transcription of plasminogen activator inhibitor-1 (PAI-1) and fibronectin (FN) in cholangiocyte [92]. They are known as HSC-activation factors. The reduction of both hinders the crosstalk between cholangiocytes and HSCs, further inhibiting the activation of HSCs and alleviating fibrosis to some extent [92].

### 5.2. Si-Wu-tang

SWT consists of four herbs: Rehmanniae Radix Praeparata (Shudi), Angelicae Sinensis Radix (Danggui), Paeoniae Radix Alba (Baishao), and Chuanxiong Rhizoma (Chuanxiong) [93]. SWT suppresses the expression of H19 by balancing bile acid homeostasis in the liver-gut axis in carbon tetrachloride induced liver fibrosis [93]. In another study, it has been determined that H19 affects the transcription of genes responsible for the cytoskeleton remodeling in HSCs and facilitates angiogenesis and hepatocytes injury by acting as a molecular sponge to absorb miR200, miR-211, and let7b [88]. Meanwhile, SWT counteracts the biological impacts mediated by H19 as summarized above. It is worth noting that there is a strong positive association observed between H19 expression and the upregulation of genes involved in pro-fibrotic and angiogenic processes in HSCs and LSECs, while a negative correlation is verified with genes responsible for protection in hepatocytes [88].

### 5.3. Danhongqing formula

Additionally, similar Traditional Chinese Medicines also include DHQ, which is composed of salvianolic acid B (Sal-B), salidroside (Salid) and artesunate (Art) and has been identified as a potent therapeutic agent [89]. DHQ inhibits the expression of H19, as well as the expression of STAT3 and subsequent phosphorylation, thereby suppressing the activation of HSCs and alleviating injury to cholangiocytes. Moreover, DHQ demonstrates inhibitory influence on the cholangiocyte proliferation and DR, which significantly delays cholestatic liver fibrosis progression [89].

The aforementioned Traditional Chinese Medicines have demonstrated unforeseen efficacy; however, whether they can confer clinical benefits to BA patients remains to be further evaluated and validated through rigorous clinical studies. Furthermore, it is imperative to conduct further research to investigate the feasibility of utilizing H19 as a therapeutic target in clinical practice. These therapeutic strategies targeting H19 are summarized in Table 1.

**Table 1.** Therapeutic strategies targeting H19.

Medicines	Models	Mechanisms	Targets	Ref
DHA	CCl4 induced male ICR mice; Primary mouse HSCs and Human LX-2 cell lines	DHA regulates lipid droplet metabolism in HSCs by inhibiting H19-induced AMPK pathway and retinoic acid signals.	AMPK pathway; retinoic acid signals; in HSCs	72, 75
CX	BDL induced C57BL/6J mice; Mouse primary cholangiocytes;Human biliary epithelial cells and Human LX-2 cell lines	CX extracts prevent bile duct proliferation *via* targeting CTCF-c-MYC-H19 pathway and c- MYC / H19 / Let-7a pathway, inhibit HSCs activation by downregulating H3K9ac-mediated and cholangiocyte-derived secretory protein PAI-1 and FN.	CTCF-c-MYC-H19; c- MYC / H19 / Let-7a; KAT2A/SMAD3-H3K9ac-PAI-1/FN; in cholangiocytes	87, 91, 92
SWT	BDL and CCl4 induced C57BL/6J mice	SWT attenuates liver fibrosis *via* regulating H19-mediated pathways involving cytoskeleton remodeling in HSCs, angiogenesis and hepatocytes injury.	miR200, miR-211, and let7b; in HSCs and hepatocytes	88, 93
DHQ	C57BL/6 Mdr2-/- mice; Cholangiocarcinoma cell line and Human LX-2 cell lines	DHQ alleviates cholestatic liver fibrosis by inhibiting H19 upregulation in cholangiocytes and H19-induced HSCs activation.	STAT3; in cholangiocytes and HSCs	89

DHA: Dihydroartemisinin; CCl4: carbon tetrachloride; HSCs: hepatic stellate cells; AMPK: AMP-activated protein kinase; CX: Chuanxiong Rhizoma; BDL: bile duct ligation; CTCF: CCCTC binding factor; H3K9ac: histone 3 lysine 9 acetylation; PAI-1: plasminogen activator inhibitor-1; FN: fibronectin; KAT2A: lysine acetyltransferase 2 A; SMAD3: mothers against decapentaplegic homolog 3; SWT: Si-Wu-Tang; DHQ: Danhongqing formula; Mdr2-/-: multidrug resistance gene 2 knockout; STAT3: signal transducer and activator of transcription 3.

## 6. Discussion

In BA, abnormal development of biliary cells exacerbates their vulnerability, making them more susceptible to viral, bacterial, and toxic assaults. The up-regulated H19, predominantly derived from damaged cholangiocytes, is efficiently transferred to the liver in the form of exosomes. Here, it is readily taken up by hepatocytes, HPCs, HSCs and macrophages, thereby regulating their responses to injury. The emergence of H19 exacerbates injury to both hepatocytes and cholangiocytes, promoting the occurrence of bile duct hyperplasia. Meanwhile, the compromised parenchymal cells exhibit the capability to propagate this signal to contiguous non-parenchymal *via* an intricate regulatory network controlled by H19, which constitutes a significant mediator in the inflammatory microenvironment of fibrosis.

Centered on H19, this review systematically synthesizes the diverse signaling pathways it modulates. Nevertheless, many of the underlying regulatory mechanisms remain to be fully elucidated. The adoption of disparate animal models, cell lines, and experimental methods across various studies may often yield divergent conclusions. What’s more, some studies employ BDL models to simulate the cholestatic process in BA. However, as BA may involve distinct pathophysiological features, this modeling approach could lead to conclusions that deviate from the actual conditions. Critically, the current understanding is predominantly based on murine models. The greater complexity of human pathophysiology means that pathways identified in mouse models may not fully apply to BA patients.

Although H19 is up-regulated and plays a core regulatory role in BA, its widespread high expression across various liver diseases may limit its utility as a diagnostic marker. Notably, recent studies have identified several herbal medicine components that can alleviate fibrosis by modulating H19 expression. Given the aggressive nature of BA, it is worth exploring whether these compounds could help retard fibrotic progression. While their clinical application will require substantial investigation, it must be acknowledged that H19 represents a highly promising target in this strategy.

## 7. Conclusions

In summary, a deeper understanding of the precise mechanisms of H19 in BA under diverse pathological conditions will facilitate its translation into clinical practice, with the ultimate goal of improving outcomes in this disease.

## Availability for data and material

Data sharing is not applicable to this article as no data were created or analysed in this study.

## References

[1] Tam PKH, Wells RG, Tang CSM, et al. Biliary atresia. Nat Rev Dis Primers. 2024;10(1):47. doi: 10.1038/s41572-024-00533-x. 38992031 PMC11956545

[2] Hartley JL, Davenport M, Kelly DA. Biliary atresia. Lancet. 2009;374(9702):1704–1713. doi: 10.1016/S0140-6736(09)60946-6. 19914515

[3] Verkade HJ, Bezerra JA, Davenport M, et al. Biliary atresia and other cholestatic childhood diseases: advances and future challenges. J Hepatol. 2016;65(3):631–642. doi: 10.1016/j.jhep.2016.04.032. 27164551

[4] Lakshminarayanan B, Davenport M. Biliary atresia: a comprehensive review. J Autoimmun. 2016;73:1–9. doi: 10.1016/j.jaut.2016.06.005. 27346637

[5] Bezerra JA, Wells RG, Mack CL, et al. Clinical and research challenges for the 21st century. Hepatology. 2018;68:1163–1173. doi: 10.1002/hep.29905. 29604222 PMC6167205

[6] Tam PKH, Yiu RS, Lendahl U, et al. Cholangiopathies - Towards a molecular understanding. EBioMedicine. 2018;36:564–393. doi: 10.1016/j.ebiom.2018.08.024. 30314899 PMC6197698

[7] Baumann U, Karam V, Adam R, et al. European liver and intestine transplant association (ELITA) and all ELTR contributing centers, prognosis of children undergoing liver transplantation: a 30-year European study. Pediatrics. 2022;150(4):e2022057424. doi: 10.1542/peds.2022-057424. 36111446

[8] Sundaram SS, Mack CL, Feldman AG, et al. Biliary atresia: indications and timing of liver transplantation and optimization of pretransplant care. Liver Transpl. 2017;23(1):96–109. doi: 10.1002/lt.24640. 27650268 PMC5177506

[9] Shen W-J, Chen G, Wang M, et al. Liver fibrosis in biliary atresia. World J Pediatr. 2019;15(2):117–123. doi: 10.1007/s12519-018-0203-1. 30465125

[10] Lorent K, Gong W, Koo KA, et al. Identification of a plant isoflavonoid that causes biliary atresia. Sci Transl Med. 2015;7(286):286ra67. doi: 10.1126/scitranslmed.aaa1652. PMC4784984 25947162

[11] Nemeth K, Bayraktar R, Ferracin M, et al. Non-coding RNAs in disease: from mechanisms to therapeutics. Nat Rev Genet. 2024;25(3):211–232. doi: 10.1038/s41576-023-00662-1. 37968332

[12] Takahashi K, Yan I, Haga H, et al. Long noncoding RNA in liver diseases. Hepatology. 2014;60(2):744–753. doi: 10.1002/hep.27043. 24493213 PMC4110118

[13] Szabo G, Bala S. MicroRNAs in liver disease. Nat Rev Gastroenterol Hepatol. 2013;10(9):542–552. doi: 10.1038/nrgastro.2013.87. 23689081 PMC4091636

[14] Li X, Liu R. Long non-coding RNA H19 in the liver-gut axis: a diagnostic marker and therapeutic target for liver diseases. Exp Mol Pathol. 2020;115:104472. doi: 10.1016/j.yexmp.2020.104472. 32454104

[15] Bartolomei MS, Zemel S, Tilghman SM. Parental imprinting of the mouse H19 gene. Nature. 1991;351(6322):153–155. doi: 10.1038/351153a0. 1709450

[16] Wang Y, Hylemon PB, Zhou H. Long noncoding RNA H19: a key player in liver diseases. Hepatology. 2021;74(3):1652–1659. doi: 10.1002/hep.31765. 33630308 PMC10071419

[17] Jiang X, Ning Q. The mechanism of lncRNA H19 in fibrosis and its potential as novel therapeutic target. Mech Ageing Dev. 2020;188:111243. doi: 10.1016/j.mad.2020.111243. 32298666

[18] Yang Z, Zhang T, Han S, et al. Long noncoding RNA H19 - a new player in the pathogenesis of liver diseases. Transl Res. 2021;230:139–150. doi: 10.1016/j.trsl.2020.11.010. 33227504 PMC9330166

[19] Shi Y, Qu F, Zeng S, et al. Targeting long non-coding RNA H19 as a therapeutic strategy for liver disease. Prog Biophys Mol Biol. 2024;194:1–9. doi: 10.1016/j.pbiomolbio.2024.09.005. 39357625

[20] Li X, Liu R, Yang J, et al. The role of LncRNA H19 in gender disparity of cholestatic liver injury in Mdr2−/− mice. Hepatology. 2017;66:869–884. doi: 10.1002/hep.29145. 28271527 PMC5570619

[21] Li X, Liu R, Huang Z, et al. Cholangiocyte-derived exosomal long noncoding RNA H19 promotes cholestatic liver injury in mouse and human. Hepatology. 2018;68(2):599–615. doi: 10.1002/hep.29838. 29425397 PMC6085159

[22] Liu R, Li X, Zhu W, et al. Cholangiocyte-derived exosomal long noncoding RNA H19 promotes hepatic stellate cell activation and cholestatic liver fibrosis. Hepatology. 2019;70(4):1317–1335. doi: 10.1002/hep.30662. 30985008 PMC6783323

[23] Li X, Liu R, Wang Y, et al. Cholangiocyte-derived exosomal lncRNA H19 promotes macrophage activation and hepatic inflammation under cholestatic conditions. Cells. 2020;9(1):190. doi: 10.3390/cells9010190. 31940841 PMC7016679

[24] Xiao Y, Liu R, Li X, et al. Long noncoding RNA H19 contributes to cholangiocyte proliferation and cholestatic liver fibrosis in biliary Atresia. Hepatology. 2019;70(5):1658–1673. doi: 10.1002/hep.30698. 31063660 PMC6819224

[25] Asai A, Miethke A, Bezerra JA. Pathogenesis of biliary atresia: defining biology to understand clinical phenotypes. Nat Rev Gastroenterol Hepatol. 2015;12(6):342–352. doi: 10.1038/nrgastro.2015.74. 26008129 PMC4877133

[26] Zhou Y, Ji H, Xu Q, et al. Congenital biliary atresia is correlated with disrupted cell junctions and polarity caused by Cdc42 insufficiency in the liver. Theranostics. 2021;11(15):7262–7275. doi: 10.7150/thno.49116. 34158849 PMC8210598

[27] Glessner JT, Ningappa MB, Ngo KA, et al. Biliary atresia is associated with polygenic susceptibility in ciliogenesis and planar polarity effector genes. J Hepatol. 2023;79(6):1385–1395. doi: 10.1016/j.jhep.2023.07.039. 37572794 PMC10729795

[28] Hellen DJ, Bennett A, Malla S, et al. Liver-restricted deletion of the biliary atresia candidate gene Pkd1l1 causes bile duct dysmorphogenesis and ciliopathy. Hepatology. 2023;77(4):1274–1286. doi: 10.1097/HEP.0000000000000029. 36645229 PMC12440248

[29] Lim YZ, Zhu M, Wang Y, et al. Pkd1l1-deficiency drives biliary atresia through ciliary dysfunction in biliary epithelial cells. J Hepatol. 2024;81(1):62–75. doi: 10.1016/j.jhep.2024.02.031. 38460793

[30] Ye C, Zhu J, Wang J, et al. Single-cell and spatial transcriptomics reveal the fibrosis-related immune landscape of biliary atresia. Clin Transl Med. 2022;12(11):e1070. doi: 10.1002/ctm2.1070. 36333281 PMC9636046

[31] Russi AE, Bezerra JA. A single-cell view of biliary atresia. Nat Rev Gastroenterol Hepatol. 2021;18(4):219–220. doi: 10.1038/s41575-021-00417-5. 33510462 PMC8201597

[32] Amarachintha SP, Mourya R, Ayabe H, et al. Biliary organoids uncover delayed epithelial development and barrier function in biliary atresia. Hepatology. 2022;75(1):89–103. doi: 10.1002/hep.32107. 34392560 PMC9983428

[33] Thorvaldsen JL, Duran KL, Bartolomei MS. Deletion of the H19 differentially methylated domain results in loss of imprinted expression of H19 and Igf2. Genes Dev. 1998;12(23):3693–3702. doi: 10.1101/gad.12.23.3693. 9851976 PMC317260

[34] Gabory A, Jammes H, Dandolo L. The H19 locus: role of an imprinted non-coding RNA in growth and development. Bioessays. 2010;32(6):473–480. doi: 10.1002/bies.200900170. 20486133

[35] Manoharan H, Babcock K, Pitot HC. Changes in the DNA methylation profile of the rat H19 gene upstream region during development and transgenic hepatocarcinogenesis and its role in the imprinted transcriptional regulation of the H19 gene. Mol Carcinog. 2004;41(1):1–16. doi: 10.1002/mc.20036. 15352122

[36] Keniry A, Oxley D, Monnier P, et al. The H19 lincRNA is a developmental reservoir of miR-675 that suppresses growth and Igf1r. Nat Cell Biol. 2012;14(7):659–665. doi: 10.1038/ncb2521. 22684254 PMC3389517

[37] Halilbasic E, Claudel T, Trauner M. Bile acid transporters and regulatory nuclear receptors in the liver and beyond. J Hepatol. 2013;58(1):155–168. doi: 10.1016/j.jhep.2012.08.002. 22885388 PMC3526785

[38] Li X, Liu R, Luo L, et al. Role of AMP-activated protein kinase α1 in 17α-ethinylestradiol-induced cholestasis in rats. Arch Toxicol. 2017;91(1):481–494. doi: 10.1007/s00204-016-1697-8. 27090119 PMC5069111

[39] Song Y, Liu C, Liu X, et al. H19 promotes cholestatic liver fibrosis by preventing ZEB1-mediated inhibition of EpCAM. Hepatology. 2017;66:1183–1196. doi: 10.1002/hep.29209. 28407375 PMC5605402

[40] Chen W, Zai W, Fan J, et al. Interleukin-22 drives a metabolic adaptive reprogramming to maintain mitochondrial fitness and treat liver injury. Theranostics. 2020;10(13):5879–5894. doi: 10.7150/thno.43894. 32483425 PMC7254999

[41] Wang C, Deng J, Deng H, et al. A novel Sox9/lncRNA H19 axis contributes to hepatocyte death and liver fibrosis. Toxicol Sci Off J Soc Toxicol. 2020;177(1):214–225. doi: 10.1093/toxsci/kfaa097. 32579217

[42] Wang Z, Yang Z, Kai J, et al. HIF-1α-upregulated lncRNA-H19 regulates lipid droplet metabolism through the AMPKα pathway in hepatic stellate cells. Life Sci. 2020;255:117818. doi: 10.1016/j.lfs.2020.117818. 32445757

[43] Sun Y, Liu C, Guo X, et al. Identification of the c-Jun/H19/miR-19/JNK1 cascade during hepatic stellate cell activation. Clin Transl Med. 2023;13(3):e1106. doi: 10.1002/ctm2.1106. 36864707 PMC9982076

[44] Tian X, Wang Y, Lu Y, et al. Conditional depletion of macrophages ameliorates cholestatic liver injury and fibrosis via lncRNA-H19. Cell Death Dis. 2021;12(7):646. doi: 10.1038/s41419-021-03931-1. 34168124 PMC8225916

[45] Wang Y, Aoki H, Yang J, et al. The role of sphingosine 1-phosphate receptor 2 in bile-acid-induced cholangiocyte proliferation and cholestasis-induced liver injury in mice. Hepatology. 2017;65(6):2005–2018. doi: 10.1002/hep.29076. 28120434 PMC5444993

[46] Nagahashi M, Takabe K, Liu R, et al. Conjugated bile acid activated S1P receptor 2 Is a key regulator of sphingosine kinase 2 and hepatic gene expression. Hepatology. 2015;61(4):1216–1226. doi: 10.1002/hep.27592. 25363242 PMC4376566

[47] Zhang L, Yang Z, Huang W, et al. H19 potentiates let-7 family expression through reducing PTBP1 binding to their precursors in cholestasis. Cell Death Dis. 2019;10(3):168. doi: 10.1038/s41419-019-1423-6. 30778047 PMC6379488

[48] Gregory PA, Bert AG, Paterson EL, et al. The miR-200 family and miR-205 regulate epithelial to mesenchymal transition by targeting ZEB1 and SIP1. Nat Cell Biol. 2008;10(5):593–601. doi: 10.1038/ncb1722. 18376396

[49] Maetzel D, Denzel S, Mack B, et al. Nuclear signalling by tumour-associated antigen EpCAM. Nat Cell Biol. 2009;11(2):162–171. doi: 10.1038/ncb1824. 19136966

[50] Banales JM, Huebert RC, Karlsen T, et al. Cholangiocyte pathobiology. Nat Rev Gastroenterol Hepatol. 2019;16(5):269–281. doi: 10.1038/s41575-019-0125-y. 30850822 PMC6563606

[51] Sato K, Marzioni M, Meng F, et al. Ductular reaction in liver diseases: pathological mechanisms and translational significances. Hepatology. 2019;69(1):420–430. doi: 10.1002/hep.30150. 30070383 PMC6324973

[52] Marakovits C, Francis H. Unraveling the complexities of fibrosis and ductular reaction in liver disease: pathogenesis, mechanisms, and therapeutic insights. Am J Physiol Cell Physiol. 2024;326(3):C698–C706. doi: 10.1152/ajpcell.00486.2023. 38105754 PMC11193454

[53] Zagory JA, Fenlon M, Dietz W, et al. PROMININ-1 promotes biliary fibrosis associated with biliary atresia. Hepatology. 2019;69(6):2586–2597. doi: 10.1002/hep.30550. 30723921 PMC6541523

[54] Short C, Zhong A, Xu J, et al. TWEAK/FN14 promotes profibrogenic pathway activation in Prominin-1-expressing hepatic progenitor cells in biliary atresia. Hepatology. 2023;77(5):1639–1653. doi: 10.1097/HEP.0000000000000026. 36626628

[55] El-Araby HA, Saber MA, Radwan NM, et al. SOX9 in biliary atresia: new insight for fibrosis progression. Hepatobiliary Pancreat Dis Int. 2021;20(2):154–162. doi: 10.1016/j.hbpd.2020.12.007. 33349604

[56] Lin Y, Zhang F, Zhang L, et al. Characteristics of SOX9-positive progenitor-like cells during cholestatic liver regeneration in biliary atresia. Stem Cell Res Ther. 2022;13(1):114. doi: 10.1186/s13287-022-02795-2. 35313986 PMC8935712

[57] Han X, Wang Y, Pu W, et al. Lineage tracing reveals the bipotency of SOX9+ hepatocytes during liver regeneration. Stem Cell Rep. 2019;12(3):624–638. doi: 10.1016/j.stemcr.2019.01.010. PMC6409431 30773487

[58] Jiang Y, Huang Y, Cai S, et al. H19 is expressed in hybrid hepatocyte nuclear factor 4α+ periportal hepatocytes but not cytokeratin 19+ cholangiocytes in cholestatic livers. Hepatol Commun. 2018;2(11):1356–1368. doi: 10.1002/hep4.1252. 30411082 PMC6211330

[59] Zhang Y, Liu C, Barbier O, et al. Bcl2 is a critical regulator of bile acid homeostasis by dictating Shp and lncRNA H19 function. Sci Rep. 2016;6(1):20559. doi: 10.1038/srep20559. 26838806 PMC4738356

[60] Lu TT, Makishima M, Repa JJ, et al. Molecular basis for feedback regulation of bile acid synthesis by nuclear receptors. Mol Cell. 2000;6(3):507–515. doi: 10.1016/s1097-2765(00)00050-2. 11030331

[61] Harari-Steinfeld R, Gefen M, Simerzin A, et al. The lncRNA H19-derived MicroRNA-675 promotes liver necroptosis by targeting FADD. Cancers (Basel). 2021;13(3):411. doi: 10.3390/cancers13030411. PMC7866230 33499244

[62] Xu Q, Deng F, Qin Y, et al. Long non-coding RNA regulation of epithelial–mesenchymal transition in cancer metastasis. Cell Death Dis. 2016;7(6):e2254. doi: 10.1038/cddis.2016.149. 27277676 PMC5143379

[63] Li X-J-Y, Zhou F, Li Y-J, et al. LncRNA H19-EZH2 interaction promotes liver fibrosis via reprogramming H3K27me3 profiles. Acta Pharmacol Sin. 2023;44(12):2479–2491. doi: 10.1038/s41401-023-01145-z. 37580495 PMC10692088

[64] Wen Y, Hou Y, Yi X, et al. EZH2 activates CHK1 signaling to promote ovarian cancer chemoresistance by maintaining the properties of cancer stem cells. Theranostics. 2021;11(4):1795–1813. doi: 10.7150/thno.48101. 33408782 PMC7778604

[65] Zhu J, Luo Z, Pan Y, et al. H19/miR-148a/USP4 axis facilitates liver fibrosis by enhancing TGF-β signaling in both hepatic stellate cells and hepatocytes. J Cell Physiol. 2019;234(6):9698–9710. doi: 10.1002/jcp.27656. 30362572

[66] Zhang L, Zhou F, Drabsch Y, et al. USP4 is regulated by AKT phosphorylation and directly deubiquitylates TGF-β type I receptor. Nat Cell Biol. 2012;14(7):717–726. doi: 10.1038/ncb2522. 22706160

[67] Trivedi P, Wang S, Friedman SL. The power of plasticity-metabolic regulation of hepatic stellate cells. Cell Metab. 2021;33(2):242–257. doi: 10.1016/j.cmet.2020.10.026. 33232666 PMC7858232

[68] Hernández-Gea V, Ghiassi-Nejad Z, Rozenfeld R, et al. Autophagy releases lipid that promotes fibrogenesis by activated hepatic stellate cells in mice and in human tissues. Gastroenterology. 2012;142(4):938–946. doi: 10.1053/j.gastro.2011.12.044. 22240484 PMC3439519

[69] Tsuchida T, Friedman SL. Mechanisms of hepatic stellate cell activation. Nat Rev Gastroenterol Hepatol. 2017;14(7):397–411. doi: 10.1038/nrgastro.2017.38. 28487545

[70] Zhang Z, Zhao S, Yao Z, et al. Autophagy regulates turnover of lipid droplets via ROS-dependent Rab25 activation in hepatic stellate cell. Redox Biol. 2017;11:322–334. doi: 10.1016/j.redox.2016.12.021. 28038427 PMC5199192

[71] Lin J, Chen A. Perilipin 5 restores the formation of lipid droplets in activated hepatic stellate cells and inhibits their activation. Lab Invest. 2016;96(7):791–806. doi: 10.1038/labinvest.2016.53. 27135793

[72] Xia S, Wang Z, Chen L, et al. Dihydroartemisinin regulates lipid droplet metabolism in hepatic stellate cells by inhibiting lncRNA-H19-induced AMPK signal. Biochem Pharmacol. 2021;192:114730. doi: 10.1016/j.bcp.2021.114730. 34400125

[73] Kluwe J, Wongsiriroj N, Troeger JS, et al. Absence of hepatic stellate cell retinoid lipid droplets does not enhance hepatic fibrosis but decreases hepatic carcinogenesis. Gut. 2011;60(9):1260–1268. doi: 10.1136/gut.2010.209551. 21278145

[74] Grumet L, Taschler U, Lass A. Hepatic Retinyl Ester Hydrolases and the Mobilization of Retinyl Ester Stores. Nutrients. 2016;9(1):13. doi: 10.3390/nu9010013. 28035980 PMC5295057

[75] Wang Z-M, Xia S-W, Zhang T, et al. LncRNA-H19 induces hepatic stellate cell activation via upregulating alcohol dehydrogenase III-mediated retinoic acid signals. Int Immunopharmacol. 2020;84:106470. doi: 10.1016/j.intimp.2020.106470. 32304991

[76] Yang J, Pi C, Wang G. Inhibition of PI3K/Akt/mTOR pathway by apigenin induces apoptosis and autophagy in hepatocellular carcinoma cells. Biomed Pharmacother. 2018;103:699–707. doi: 10.1016/j.biopha.2018.04.072. 29680738

[77] Huang T-J, Ren J-J, Zhang Q-Q, et al. IGFBPrP1 accelerates autophagy and activation of hepatic stellate cells via mutual regulation between H19 and PI3K/AKT/mTOR pathway. Biomed Pharmacother. 2019;116:109034. doi: 10.1016/j.biopha.2019.109034. 31152924

[78] Yang S, Chang N, Li W, et al. Necroptosis of macrophage is a key pathological feature in biliary atresia via GDCA/S1PR2/ZBP1/p-MLKL axis. Cell Death Dis. 2023;14(3):175. doi: 10.1038/s41419-023-05615-4. 36859525 PMC9977961

[79] Lages CS, Simmons J, Maddox A, et al. The dendritic cell-T helper 17-macrophage axis controls cholangiocyte injury and disease progression in murine and human biliary atresia. Hepatology. 2017;65(1):174–188. doi: 10.1002/hep.28851. 27641439 PMC5191928

[80] Nagayabu K, Fumino S, Shimamura A, et al. The clinical impact of macrophage polarity after Kasai portoenterostomy in biliary atresia. Front Pediatr. 2024;12:1338131. doi: 10.3389/fped.2024.1338131. 38318455 PMC10839051

[81] Zhu Y, Ni T, Lin J, et al. Long non-coding RNA H19, a negative regulator of microRNA-148b-3p, participates in hypoxia stress in human hepatic sinusoidal endothelial cells via NOX4 and eNOS/NO signaling. Biochimie. 2019;163:128–136. doi: 10.1016/j.biochi.2019.04.006. 31082428

[82] DeLeve LD. Liver sinusoidal endothelial cells in hepatic fibrosis. Hepatology. 2015;61(5):1740–1746. doi: 10.1002/hep.27376. 25131509 PMC4333127

[83] Lee H-C, Chang T-Y, Yeung C-Y, et al. Genetic variation in the vascular endothelial growth factor gene is associated with biliary atresia. J Clin Gastroenterol. 2010;44(2):135–139. doi: 10.1097/MCG.0b013e3181b152c2. 19713864

[84] Edom PT, Meurer L, da Silveira TR, et al. Immunolocalization of VEGF A and its receptors, VEGFR1 and VEGFR2, in the liver from patients with biliary atresia. Appl Immunohistochem Mol Morphol. 2011;19(4):360–368. doi: 10.1097/PAI.0b013e3182028a8e. 21285868

[85] Mariotti V, Fiorotto R, Cadamuro M, et al. New insights on the role of vascular endothelial growth factor in biliary pathophysiology. JHEP Rep. 2021;3(3):100251. doi: 10.1016/j.jhepr.2021.100251. 34151244 PMC8189933

[86] Quelhas P, Breton MC, Oliveira RC, et al. HIF-1alpha-pathway activation in cholangiocytes of patients with biliary atresia: an immunohistochemical/molecular exploratory study. J Pediatr Surg. 2023;58(3):587–594. doi: 10.1016/j.jpedsurg.2022.08.020. 36150932

[87] Li Y, Li F, Ding M, et al. Chuanxiong Rhizoma extracts prevent liver fibrosis via targeting CTCF-c-MYC-H19 pathway. Chin Herb Med. 2024;16(1):82–93. doi: 10.1016/j.chmed.2023.07.003. 38375042 PMC10874761

[88] Qu J, Xue X, Wang Z, et al. Si-Wu-Tang attenuates liver fibrosis via regulating lncRNA H19-dependent pathways involving cytoskeleton remodeling and ECM deposition. Chin J Nat Med. 2024;22(1):31–46. doi: 10.1016/S1875-5364(24)60560-1. 38278557

[89] Li M, Zhou Y, Zhu H, et al. Danhongqing formula alleviates cholestatic liver fibrosis by downregulating long non-coding RNA H19 derived from cholangiocytes and inhibiting hepatic stellate cell activation. J Integr Med. 2024;22(2):188–198. doi: 10.1016/j.joim.2024.03.006. 38472011

[90] Freschi A, Del Prete R, Pignata L, et al. The number of the CTCF binding sites of the H19/IGF2: IG-DMR correlates with DNA methylation and expression imprinting in a humanized mouse model. Hum Mol Genet. 2021;30(16):1509–1520. doi: 10.1093/hmg/ddab132. 34132339 PMC8330897

[91] Li Y, Liu R, Li J, et al. Senkyunolide A interrupts TRAF6-HDAC3 interaction to epigenetically suppress c-MYC and attenuate cholestatic liver injury. J Adv Res. 2026;79:935–951. doi: 10.1016/j.jare.2025.04.002. 40187727 PMC12766224

[92] Li Y, Ma Z, Ding M, et al. Chuanxiong Rhizoma extracts prevent cholestatic liver injury by targeting H3K9ac-mediated and cholangiocyte-derived secretory protein PAI-1 and FN. Chin J Nat Med. 2023;21(9):694–709. doi: 10.1016/S1875-5364(23)60416-9. 37777319

[93] Xue X, Wu J, Ding M, et al. Si-Wu-Tang ameliorates fibrotic liver injury via modulating intestinal microbiota and bile acid homeostasis. Chin Med. 2021;16(1):112. doi: 10.1186/s13020-021-00524-0. 34736501 PMC8570021

